# A Dermonutrient Containing Special Collagen Peptides Improves Skin Structure and Function: A Randomized, Placebo-Controlled, Triple-Blind Trial Using Confocal Laser Scanning Microscopy on the Cosmetic Effects and Tolerance of a Drinkable Collagen Supplement

**DOI:** 10.1089/jmf.2019.0197

**Published:** 2020-01-31

**Authors:** Sabrina Laing, Stephan Bielfeldt, Carolin Ehrenberg, Klaus-Peter Wilhelm

**Affiliations:** proDERM Institute for Applied Dermatological Research, Schenefeld-Hamburg, Germany.

**Keywords:** antiaging, collagen peptides, dermal collagen structure, dermonutrient, oral collagen supplement, skin beauty

## Abstract

The purpose of this randomized, placebo-controlled, triple-blind trial on 60 healthy female volunteers was to assess the cosmetic effects on skin quality of a food supplement containing special collagen peptides together with acerola extract, vitamin C, vitamin E, biotin, and zinc after an intake of 12 weeks (Elasten^®^, QUIRIS Healthcare, Germany). To reduce assessment bias maximally and increase the accuracy and objectivity of the outcomes, the trial design was triple blinded in a manner that neither the subjects nor the person administering the products nor the person who assessed the primary outcomes knew which subjects had received the test product and which had received the placebo. The expert grader assessing the confocal laser scanning microscopy images was additionally blinded regarding the time when the image was taken (on days 1 or 85). The objective, blinded, and validated image analyses using confocal laser scanning microscopy showed a significant improvement of the collagen structure of facial skin (primary endpoint) after intake of the test product, while no improvements were found after intake of the placebo. The proven positive nutritional effect on the collagen structure was fully consistent with positive subjective evaluations of relevant skin parameters such as elasticity, crinkliness/wrinkliness, and evenness in different body areas such as face, hands, décolleté, neck, backside, legs, and belly, all serving as secondary endpoints. The test product was found to be safe and very well tolerated. A cosmetically relevant improvement of the facial skin was demonstrated after administration of the collagen supplement.

## Introduction

The environment as well as stress and chronological aging can induce irreversible, and also reversible, changes in the structural components of the skin that lead to visible signs of aging such as increased dryness and wrinkle formation.^1–3^ The appearance of the skin depends to a large extent on the homeostasis of the dermal extracellular matrix that is primarily defined by collagen and similar fibers.^4^ Orally administered collagen peptides and synergistically acting dermonutrients can reach the deeper layer of the skin and stimulate the endogenous production of extracellular matrix components in the skin.^5^ These oligopeptides are derived from an enzymatic hydrolysis leading to highly bioavailable and thus bioactive short-chain collagen peptides that are able to induce beneficial cosmetic effects.^6^ The purpose of this study was to investigate the cosmetic effect of a collagen supplement on the appearance of skin in healthy women.

## Materials and Methods

### Study design and ethical aspects

The study was performed using a randomized, parallel group, placebo-controlled, and double-blind design following the principles of Good Clinical Practice. The study was registered in the German Clinical Trial Register and the ICTRP (no. DRKS00017093). The investigation was in full compliance with the principles outlined in the Declaration of Helsinki and with national regulations. A written declaration of informed consent was received from all volunteers. The trial protocol was approved by the Freiburg Ethics Committee International on December 18, 2017.

### Study participants

A total of 60 female trial participants ages between 40 and 70 years were enrolled in this study. All participants finished the study without major protocol deviations. The main exclusion criteria were active skin diseases at the measurement area, systemic therapy with immunosuppressive drugs, antiphlogistic agents, analgesic drugs, antihistamines or antibiotics, or topical medication at the measurement site before the start of the study and/or throughout the entire course of the study, and intensive use of skin care products at the measurement site within 1 month before the start of the study.

Participants were instructed not to apply any leave-on or decorative cosmetics on the measurement site in the morning before the start of the study, to maintain their living habits, and not to begin or change any estrogen or progesterone therapy. Any intake of other products, which may have an effect on the skin during the study, was not allowed.

### Study schedule

Eighty-four units (drinking ampoules) of the study product (25 mL per unit) were provided to each participant. The products were to be taken by the subjects at home, once a day before or together with a meal, for 12 weeks. Assessment times were the day before the first intake (day 1) and the day after 12 weeks of intake (day 85). Compliance and tolerability were monitored on days 29, 57, and 85. Efficacy data were collected at the end of the study.

### Test materials

The study product is classified as a food supplement. Both the test product and placebo were delivered in identical drinking ampoules. The test product (Elasten^®^, QUIRIS Healthcare) contained 2.5 g specific short-chain collagen oligopeptides, 666 mg acerola fruit extract, 80 mg vitamin C, 3 mg zinc citrate, 2.3 mg vitamin E, and 50 *μ*g biotin. Additional ingredients also contained in the placebo preparation included potassium sorbate, sodium benzoate, carboxymethylcellulose, citric acid, natural aroma, and water. The placebo did not contain any nutrients. Test product and placebo were randomly assigned to two equally sized study arms (30 participants in each).

### Confocal microscopy

The collagen structure (*i.e.*, the fragmentation of collagen in the reticular dermis) was assessed by confocal laser scanning microscopy (VIVASCOPE 1500; Lucid, Inc., Rochester, NY, USA), which allows the cellular microstructure to be visualized in 5 *μ*m horizontal optical cross sections of the epidermis and the dermis with a maximal depth of 200 *μ*m.^7^ The skin can be imaged *in vivo* in its native state without further preparation. This method enables an *in vivo* mapping of the skin since the different microstructures within the skin cause natural variations of the refraction index. For example, cytoplasm with a refraction index close to that of water (reflectance index 1.33) is depicted with a very low contrast. Melanin and keratin (reflectance index 1.7) have a higher refraction index and thereby act as natural contrast agents. The optical section resolution is 5 *μ*m with an image size of 500 × 500 *μ*m. The test area was also captured with a macrocamera, which allowed correlated confocal and photographic images. All measurements were done on the cheeks after an acclimatization period of at least 30 min in a climatized room (21 ± 1°C and 50 ± 5% relative humidity) under highly standardized conditions. If necessary, facial hairs were carefully removed with a clipper by a technician 30 min before the measurement was taken.

### Collagen structure

The collagen structure, the appearance of which is determined by the relationship between reticulated, coarse, huddled, and curled collagen fibers, changes with age. Young-looking healthy skin mostly shows thin reticulated fibers. With aging, the proportion of thin reticulated fibers progressively decreases, in favor of coarse and huddled collagen fibers. In addition, the proportion of bright curled fibers progressively increases in older people.^8^

**FIG. 1.**
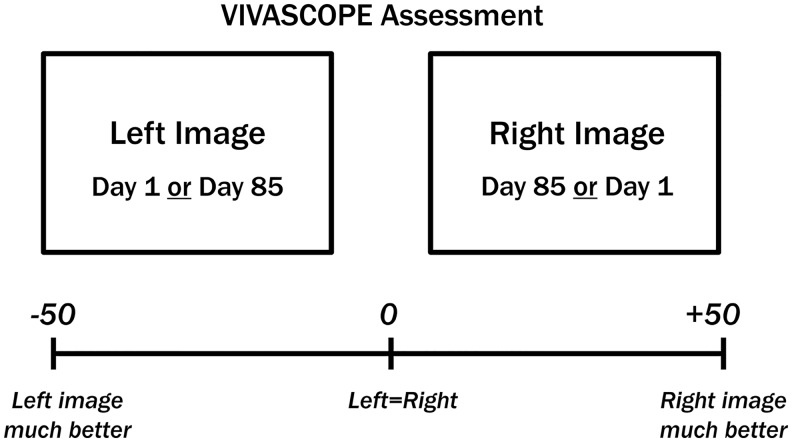
Assessment of the appearance of the collagen structure by expert graders using images of confocal laser scanning microscopy (VIVASCOPE).

Such changes are clearly recognizable in confocal images. With age, the dermal collagen network becomes increasingly fragmented, which is characterized by shorter and less organized fibers and accumulating degraded collagen fragments. The younger and healthier the skin, the larger, more diffuse, and less fragmented are the collagen structures. Differences between confocal images taken before and after the intervention were assessed by an expert grader, based on the degree of collagen fragmentation. The collagen structure was assessed using at least three uniformly distributed stacks over the measurement area in the face, starting within the stratum corneum with up to 40 repetitions at 5 *μ*m scanning steps. Confocal images targeting the area of the collagen network were chosen for analysis. Images taken on days 1 and 85 were compared on a computer screen in a validated, blinded, and left/right randomized manner by the expert grader on a visual analog scale (VAS) from −50 ( = left image better) to +50 ( = right image better) (Fig. 1). For details of the blinding procedure, see chapter 2.8.

### Subjective evaluation

Subjective assessments of product traits and cosmetic effectiveness on the quality of the skin were recorded on day 85 using a questionnaire. The general evaluation of product effects on the skin was covered using 15 questions/statements concerning the improvement of the following criteria: dryness, scaliness, elasticity, complexion, firmness, crinkliness, radiance, evenness, softness, healthiness and suppleness of the skin, the need for external care products, the perception of a high product effect, the perception of a strong product effect, the evaluation of skin changes noticed by people in the participants` personal environment, and the general satisfaction with the product. Furthermore, the positive product effects (less dry, fewer wrinkles/scales, more radiant/even/supple, softer, healthier, firmer) were related to one or more of the body areas (face, hands, décolleté, neck, backside, legs, belly) at the end of the study.

The tolerability and digestibility of the product, as well as the sensitivity of the skin, were also assessed at the end of the study. Adverse events or severe adverse events and changes in concomitant therapy, as well as compliance checks, had to be documented on days 29, 57, and 85.

### Blinding

The triple-blinded procedure was chosen to maximally reduce assessment bias and to increase the accuracy and objectivity of the outcomes. The trial design was triple-blinded in a manner that neither the subject nor the person administering the products nor the person who assessed the primary outcomes knew which subjects had received the test product and which had received a placebo. The expert grader of the confocal laser scanning microscopy images was additionally blinded regarding the time when the image was taken (on days 1 or 85).

### Statistical analyses

Safety and tolerance data listing was based on the safety population (intention to treat population), which included all trial participants who were enrolled into the study and who received at least the first dose of the study intervention. The efficacy analysis was based on the per-protocol population, including all subjects randomized in the study in accordance with both the inclusion and exclusion criteria and who finished the study according to the protocol without any major protocol violations and who had not withdrawn their consent.

The primary endpoint of the study was defined as the change in the collagen structure. The mean value of at least three image gradings per subject was used as raw data for further statistical analyses and comparison of placebo and verum. For the descriptive data analysis of the collagen structure, number of individuals (N), means, standard deviations, and 95% confidence intervals (CI) of all subjects were calculated. Bar charts with mean values and 95% CIs were created to compare the placebo and verum interventions. For the primary analysis, a *t*-test for independent samples with a significance level of *α* = 0.05 was conducted on the raw data.

The secondary endpoint was the trial participants' subjective assessment of product effects on the quality of the skin by the subjects enrolled in this controlled study. Within the context of a descriptive data analysis, a significance level of 0.05 (*α*) was chosen for statistical analysis. Counts, percentages, and means of scores were provided for all parameters of the questionnaire. For the parameter collagen structure, pairwise comparisons of postintervention assessment and baseline were performed on the raw data for each intervention separately with paired *t*-tests versus the benchmark of 0 (no differences between assessment times). The parameters of the subjective assessments serving as the secondary endpoint were analyzed using the Mann–Whitney–Wilcoxon test for pairwise comparison of the study groups.

Statistical analyses were performed according to the principles of the ICH guideline E9 “Statistical Principles for Clinical Trials” using the SAS software, version 9.4 for Windows, SAS Institute, Inc., Cary, NC, USA.

## Results

### CONSORT flow diagram of the controlled interventional trial

**FIG 2.**
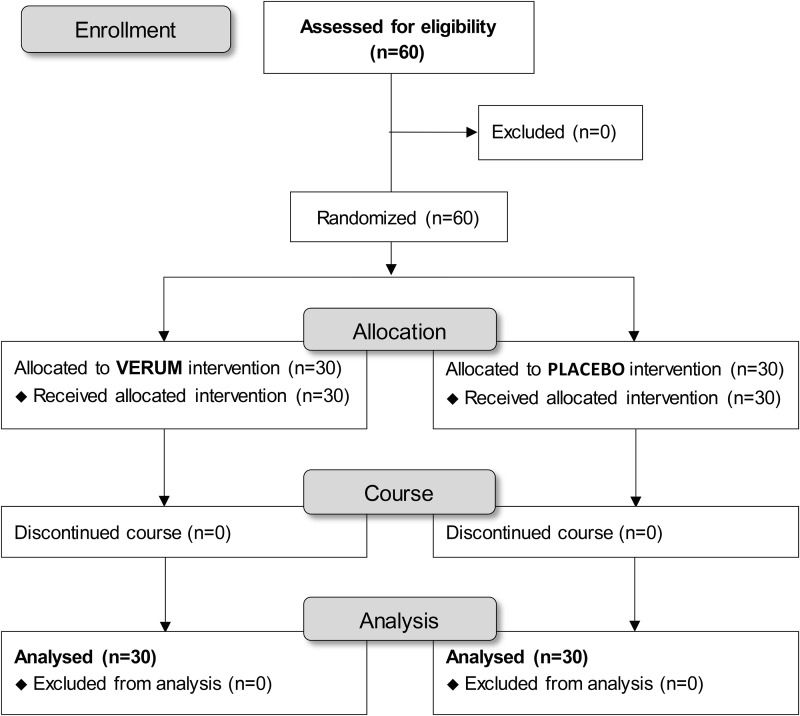
Recruitment of eligible subjects with intervention protocol and assessment.

Sixty trial participants with a mean age of 54.4 ± 7.5 years were statistically analyzed. No trial participants had to be excluded during screening or at any point in the study. The study population and the per-protocol population were identical (*n* = 60). The flow of subjects through the controlled interventional trial is depicted in a CONSORT (Consolidated Statement of Reporting Trials) conform diagram (Fig. 2).

The study was conducted as a two-arm interventional trial of the test product against placebo for 12 weeks. All enrolled subjects were examined before the first intake of test product or placebo (day 1) and after 12 weeks of intake of test product or placebo (day 85). The safety and efficacy of the test product compared with placebo could be confirmed for the entire study population.

### Analyses of collagen structure by confocal microscopy

**FIG. 3.**
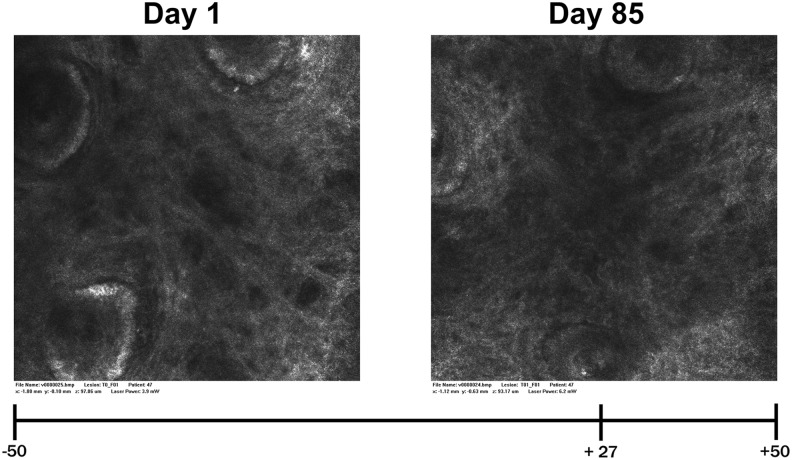
VIVASCOPE images (subject RD47) showing the collagen structure of the subcutis, taken before (*left*) and after (*right*) 85 days of intake of the test product; VAS at the *bottom*. The blinded expert grader assessed the *right* image clearly better than the *left* image with a score of +27 (0 = no difference). VAS, visual analog scale.

The application of the confocal laser scanning microscopy procedure provides the opportunity to visualize the collagen structure in the subcutaneous tissue *in vivo*. This method allows a demonstration of the influence of an oral collagen supplement not only on skin characteristics but also especially on the presence of collagen fibers as the most important structural protein of the skin. With the help of a blinded expert grader analyzing such randomly presented microscopy images taken before and after 12 weeks of supplementation of placebo and the test product, a significant improvement of the collagen structure of the facial skin could be demonstrated in response to the test product. Figure 3 shows the different appearances of the collagen structure on day 1 and on day 85 using the example of a single subject (RD47) from the active (verum) intervention group: the evaluation of the collagen structure (*i.e.*, degree of collagen fragmentation) for day 85 (right image) was considerably improved, reflected by an expert rating of +27 within the VAS range of between −50 (left image much better) and +50 (right image much better).

**FIG. 4.**
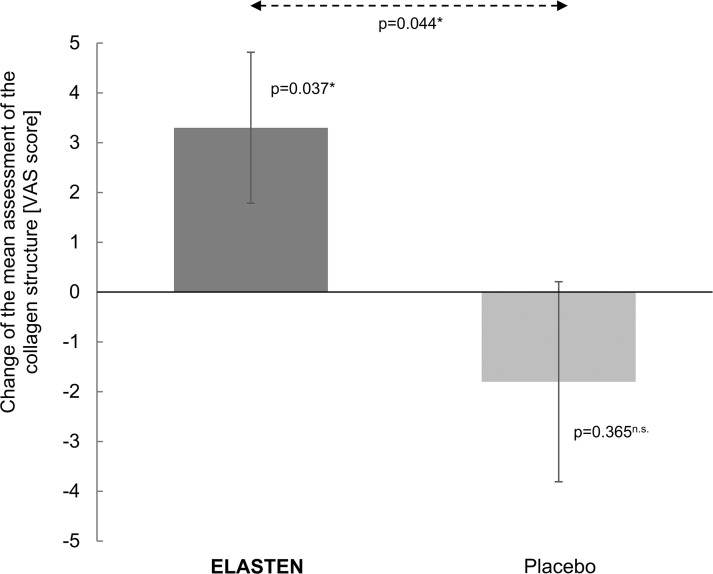
Appearance of the collagen structure by VIVASCOPE—bar chart with mean values and 95% confidence intervals (test product Elasten^®^: *n* = 30, placebo: *n* = 30), VAS: −50 ( = day 1 better) to +50 ( = day 85 better) (0 = no difference); ^n.s.^not significant, *significant (*P* ≤ .05).

Both the intragroup changes and the intergroup differences confirmed the positive effect of the test product on the human skin. The collagen structure and therefore the quality of the dermis were significantly improved on day 85 compared with benchmark 0 on day 1 after intake of the test product. In contrast, no significant difference to benchmark 0 was detected for placebo. As such, the comparison of treatments revealed a significantly better outcome for the test product than for placebo. Figure 4 demonstrates the improvement of the facial collagen structure by the test product compared with placebo. Intake of placebo resulted in a slight but not significant deterioration of the collagen structure on day 85 compared with day 1.

### Analyses of subjective assessments (secondary endpoints)

The analyses of the questionnaire concerning the cosmetic effectiveness of the products revealed a clear overall preference of the test product.

**FIG. 5.**
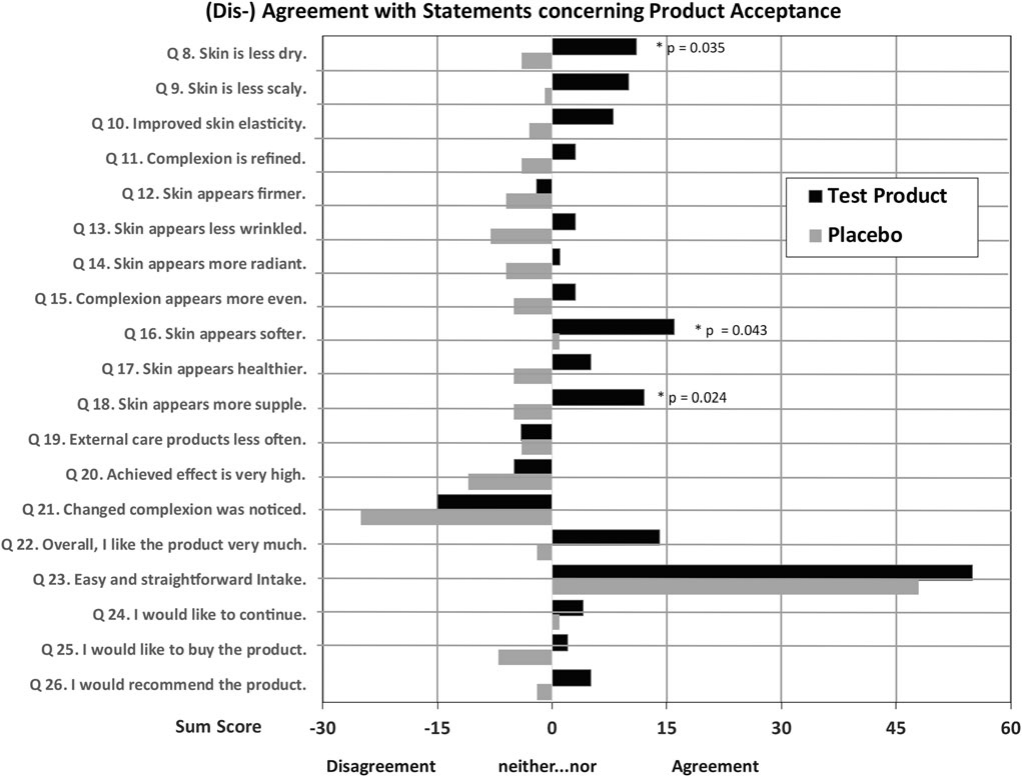
Results of questionnaire regarding product acceptance on day 85, sum of single scores (“2” = I completely agree; “1” = I agree; “0” = neither…nor; “−1” = I disagree; “−2” = I completely disagree) symbolizing agreement (positive values) or disagreement (negative values) with the product, *black bars* = test product, *gray bars* = placebo, *P* values shown where differences are significant (*P* ≤ .05, one-sided Mann–Whitney–Wilcoxon test).

The superiority of the test product compared with placebo becomes clearer through summation of the single scores (“2” = I completely agree; “1” = I agree; “0” = neither…nor; “−1” = I disagree; “−2” = I completely disagree). A higher sum score with a better evaluation for the test product was found (Fig. 5) for all criteria, with the exception of one criterion (Q19) “external care products less often,” with no difference between test product and placebo. The differences in favor of the test product are significant for items regarding skin moisture, suppleness, and softness of skin after product intake. Trial participants noted a superior effect of the test product compared with placebo in all named areas of the body. The best subjective ratings in favor of the test product were found for the backside, legs, neck, and belly, with the most frequently named improvements being “less dry,” “fewer wrinkles,” “more even,” and “more supple.”

### Tolerability

The tolerance toward the tested products was evaluated very positively by all the trial participants. No adverse effects were reported. The test product was safe and very well tolerated.

## Discussion

This randomized, placebo-controlled, triple-blind trial demonstrated the cosmetic effects of a food supplement with special collagen peptides and other dermonutrients on skin quality and collagen structure by means of confocal laser scanning microscopy.

Reduction of assessment bias is essential and increases the accuracy and objectivity. This was achieved by randomization and triple-blinding, through which the treatment modality was unknown to (1) the trial participant, (2) the persons who administered the products, and (3) the person who assessed the outcomes. The expert grader of the confocal laser scanning microscopy images was also blinded regarding the time when the image was taken (pre/post treatment).

Ingestion of collagen peptides can increase collagen synthesis but differ significantly with regard to molecular size and amino acid composition.^5–7,9–11^ The specific bovine collagen peptides of the [HC]-collagen-complex are characterized by a high homogeneity of the amino acid profile with the human collagen protein. Previous peptide mass fingerprinting and liquid chromatography-based proteomic methods have demonstrated a high level of sequence identity of bovine and porcine collagen with human skin collagen.^12^ Small cryptic peptides called cryptides together with specific cyclopeptides and matrikines generated from collagen biodegradation and bioresorption can enhance collagen synthesis in humans and can be successfully used to improve, sustain, and maintain skin regeneration, renewal, and repair throughout the entire body.^5,13^

A high bioavailability as assured by the specific digestion of the oligopeptides to the even shorter bioactive di- and tripeptides formed in the gastrointestinal tract supplies the skin with the specific collagen I and III protein amino acid pattern needed to sustain a sufficient synthesis of the decisive component of the skin biomatrix.^5–7,10–12^ The collagen structure also requires specific cofactors such as vitamin C that are needed for the formation of collagen as well as antioxidants that protect the protein from oxidation.^14,15^ Since aging, stress, and pollution reduce collagen synthesis and thereby negatively impact skin appearance, sufficient nutritional support is a decisive factor.^1,5,6^ The results of this interventional trial demonstrate the measurable cosmetic effectiveness of the test product in improving skin quality. The findings are in line with those of previously published studies investigating the effects of oral collagen supplementation on the dermal collagen network.^5–7^ The measured positive changes in the structure of connective tissue are clearly associated with functional improvements as reflected by the clear and convincing effects noted in the subjective ratings of the study participants.

This randomized, placebo-controlled, triple-blind trial confirmed the positive cosmetic effects of the collagen-containing food supplement on the quality and appearance of the skin.
